# Thermal, Viscoelastic, Mechanical and Wear Behaviour of Nanoparticle Filled Polytetrafluoroethylene: A Comparison

**DOI:** 10.3390/polym12091940

**Published:** 2020-08-27

**Authors:** Levente Ferenc Tóth, Patrick De Baets, Gábor Szebényi

**Affiliations:** 1 Soete Laboratory, Department of Electromechanical, Systems and Metal Engineering, Ghent University, Technologiepark Zwijnaarde 46, B-9052 Zwijnaarde, Belgium; levente.toth@ugent.be (L.F.T.); patrick.debaets@ugent.be (P.D.B.); 2 Department of Polymer Engineering, Faculty of Mechanical Engineering, Budapest University of Technology and Economics, Műegyetem rkp. 3, H-1111 Budapest, Hungary; 3 FlandersMake@UGent – Core lab EEDT-MP, Technologiepark-Zwijnaarde 131, B-9052 Zwijnaarde, Belgium

**Keywords:** nanoparticle filled PTFE, mechanical testing, thermal properties, sliding wear

## Abstract

In this research work, unfilled and mono-filled polytetrafluoroethylene (PTFE) materials were developed and characterised by physical, thermal, viscoelastic, mechanical, and wear analysis. The applied fillers were graphene, alumina (Al_2_O_3_), boehmite alumina (BA80), and hydrotalcite (MG70) in 0.25/1/4/8 and 16 wt % filler content. All samples were produced by room temperature pressing–free sintering method. All of the fillers were blended with PTFE by intensive dry mechanical stirring; the efficiency of the blending was analysed by Energy-dispersive X-ray spectroscopy (EDS) method. Compared to neat PTFE, graphene in 4/8/16 wt % improved the thermal conductivity by ~29%/~84%/~157%, respectively. All fillers increased the storage, shear and tensile modulus and decreased the ductility. PTFE with 4 wt % Al_2_O_3_ content reached the lowest wear rate; the reduction was more than two orders of magnitude compared to the neat PTFE.

## 1. Introduction

Nanoparticle filled thermoplastics are widely investigated materials due to their beneficial features. These nanoparticles can enhance the mechanical, thermal properties and flame retardancy of the thermoplastics and they can achieve a significant improvement of wear resistance as well [1,2,3,4,5]. Focusing on polytetrafluoroethylene (PTFE), this thermoplastic has high thermal stability, excellent chemical resistance, low coefficient of friction, and good self-lubricating property compared to other semi-crystalline thermoplastics. Well-known limitations of PTFE are the relatively low mechanical properties and the high wear rate, which can be improved with the application of reinforcements such as fibers and micro- or nanoparticles [6,7]. The need for improved mechanical properties comes from those application areas where PTFE is applied as a matrix material [8]. Sliding bearings can be an example, where the surpassing of the mechanical performance of neat PTFE is a requirement.

It is well known in the literature that graphene and alumina (Al_2_O_3_) nanofillers can improve the wear resistance of PTFE by 2 to 3 orders of magnitude [9,10,11]. In literature, it is hypothesised that PTFE molecular chains are subjected to mechanical chain scission during the wear process, forming carboxyl functional groups (–COOH) at the end of the broken PTFE molecular chains under the action of air and humidity [10,12]. These in situ formed carboxyl functional groups of PTFE can participate in complex formation with the alumina nanoparticles improving the wear resistance [12]. Besides the above-mentioned relevant mechanism, the wear resistance is affected by the physical, thermal, mechanical, and morphological properties of the materials of interest. In literature, a comprehensive investigation of the tribological characterisation of nanoparticle filled PTFE can be found, but a detailed material characterisation is hardly available, and as a result, we lack a thorough understanding of this hybrid material.

Boehmite alumina (AlO(OH), BA80) and hydrotalcite (Mg_6_Al_2_(CO_3_)(OH)_16_·4(H_2_O), MG70) are also potential filler candidates in thermoplastic matrices. They have a high number of functional groups, which can be promising to increase the wear-resistance of thermoplastics as they can initiate a higher number of complex formation due to their extra functional groups [13,14]. Karger-Kocsis et al. reported in their review that boehmite improves the Young`s modulus, toughness, creep resistance, and thermal stability of polymer matrices [14,15]. With respect to flame retardancy, boehmite can reduce the smoke generation and the peak of heat release rate [14]. These results were achieved in many different thermoplastic matrices such as in high-density polyethylene (HDPE), ultrahigh molecular weight polyethylene (UHMWPE), low-density polyethylene (LDPE), polypropylene (PP) or polystyrene (PS) [14]. The functionalisation of other fillers, e.g., carbon graphene/nanotubes, also shows promising results [16]. In the base of the introduced literature survey, in this research work it is hypothesized that in PTFE matrix material, the functional groups of BA80 and MG70 nanofillers can participate in complex formation with the in situ “functionalized” PTFE carboxyl groups. As a result, a more durable and adequate transfer layer can be formed, which can enhance the wear resistance. BA80 and MG70 fillers, in combination with PTFE, have not been investigated yet; in this way, the potential effect of their extra functional groups on the wear resistance is still a question.

The present research work is focusing on the physical, thermal, viscoelastic, mechanical, and wear characterisation of nanoparticle-filled PTFE. Regarding these materials, there are two main purposes. The first is a comprehensive material characterisation, which can be an added value for the existing tribological researches of graphene/Al_2_O_3_ filled PTFE. The other aim is the investigation of novel BA80/MG70-filled PTFE, comparing them to graphene/Al_2_O_3_-filled samples.

## 2. Materials and Methods

### 2.1. Materials

The used polytetrafluoroethylene (PTFE) powder was 3M^TM^ Dyneon^TM^ TFM^TM^ 1700 with ~25 µm average particle size, produced by the 3M Company (Minnesota Mining and Manufacturing Company, Maplewood, MN, USA). The applied graphene was xGnP^®^ Graphene Nanoplatelets Grade M from XG Sciences (Lansing, MI, USA). The average particle size was ~25 µm. The applied 1015WW alpha alumina (Al_2_O_3_) with 99.5% purity was produced by Nanostructured & Amorphous Materials Inc. (Houston, TX, USA). The average nanoparticle size was between 27 and 43 nm. The applied boehmite alumina (AlO(OH)) was Disperal^®^ 80 (BA80) from Sasol (Johannesburg, South Africa) with 35 µm average particle size and 80 nm average crystallite size. Al_2_O_3_ content of BA80 was 80%. Pural^®^ MG70 hydrotalcite (MG70) from Sasol (Johannesburg, South Africa) has a double-layered metal hydroxide structure including magnesium and aluminium hydroxides (70:30, respectively). It had ~45 µm average particle size.

### 2.2. Production Protocol and Properties of the Unfilled/Filled PTFE Samples

The composition of the produced PTFE-based materials can be seen in Table 1. The applied production technique was room temperature pressing–free sintering method. PTFE and filler powders were blended by intensive dry mechanical stirring, which is a less hazardous and more environment-friendly alternative than solvent blending method. Stirring was provided by a rotating blade grinder (180 W power); the stirring time was 30 s. The pressing was carried out with a Zwick Z250 universal tester at room temperature. The pressing speed was 2 mm/min until reaching 12.5 MPa pressure. Subsequently, 3 min of dwelling time was held at the same level of pressure. After the 3 min of dwelling time, the mould was unloaded, removed from the universal testing machine, and the pressed ‘green’ material was ready for the sintering (heating) process. The sintering procedure was carried out in an oven, in air atmosphere. The sintering cycle was the following: 90 °C/h heating rate from room temperature to 370 °C, 2 h dwelling time in 370 °C maximal temperature and 30 °C/h cooling rate. Alumina and MG70 filled samples were developed only with 1 and 4 wt % filler content because according to some previous TGA measurements, these fillers cause a thermal instability during the sintering process in higher filler content. As graphene has a high volume ratio, PTFE filled with 0.25 wt % was also investigated.

Disc-shaped samples were pressed with 10/120 mm diameter and 4 mm thickness. Specimens with 10 mm diameter were used for compressive tests, and all other specimens were cut out or milled from the samples with 120 mm diameter. The results of repeatability tests are introduced with the mean and standard deviation values related to ±1σ.

### 2.3. Material Characterisation

#### 2.3.1. Spectroscopy and Micro-Analyses

Raman spectrometry was carried out with a Horiba Jobin Yvon Labram 300 spectrometer (Horiba, Kyoto, Japan) equipped with charge-coupled device (CCD) detector and 532 nm Nd-YAG LASER. The grating was 1800 grooves/mm. The investigated spectrum range was between 1789 and 346 cm^−1^ in wavenumbers.

Energy-dispersive X-ray spectroscopy (EDS) investigations were carried out with a JEOL JSM 6380LA device (JEOL, Tokyo, Japan) with 15 kV accelerating voltage, 10 sweep counts and 0.1 msec dwell time. The sufficient electron conductivity of the samples was provided by sputtering of the surface with gold (Au) in a JEOL FC-1200 device. Micrographs were taken in high vacuum mode.

#### 2.3.2. Density Measurement

The density of the sintered samples was estimated by immersion method (ISO 1183-1:2012). The mass of the samples was measured first in air, and afterwards in ethanol. The mean and standard deviation values were calculated from at least five measurements.

#### 2.3.3. Thermal Conductivity

The thermal conductivity of the unfilled/filled PTFE was measured by a thermal conductivity measurement device, developed at the Department of Polymer Engineering of BME (Budapest, Hungary) [17,18]. The measurements were carried out according to the transient hot plate method. The measured sample is mounted between two 80 mm × 80 mm sized copper plates; the upper one is heated by aluminium-chromium (AlCr) heating wire, while the lower one is cooled by four Peltier cells. The temperature was registered by 2-2 built-in NTC thermistors (Epcos B57045K) at the upper and lower sides. The applied test temperature was 50 °C. The mean and standard deviation values were calculated from at least five measurements.

#### 2.3.4. Mechanical Analyses

Dynamic mechanical analysis (DMA) was carried out with a TA Instruments DMA Q800 device (TA Instruments, New Castle, DE, USA) in multi-frequency-strain mode. Three-point bending configuration was applied, the distance between the supports was 20 mm. The applied temperature range was between −120 and 330 °C with 3 °C/min heating rate and 1 Hz frequency. The isothermal dwelling time at −120 °C was 5 min. The applied oscillation strain was 0.05% with 6 N static force.

Hardness measurements were carried out with a Zwick H04.3150.000 digital hardness tester (Zwick Roell Group, Ulm, Germany) in Shore-D measurement range.

The compressive properties of the filled/unfilled PTFE samples were measured by a Zwick Z020 universal tester (Zwick Roell Group, Ulm, Germany) equipped with a 20 kN load cell. The tested cylindrical samples had 10 mm diameter and 10 mm height. The crosshead speed during the test was 2 mm/min.

The shear tests were carried out with the use of Iosipescu shear test method set up. All tests were run according to ASTM D 5379-05 standard. The strain measurement was performed with a Digital Image Correlation (DIC) measurement system (Mercury Monet with 5 MP camera). The shear properties of the polymer samples were measured by a Zwick Z020 universal tester equipped with a 20 kN load cell. The crosshead speed during the test was 2 mm/min. With this test method, the shear properties of materials can be determined by the use of V-notched beams (4 mm notch depth on each side). The samples had 80 mm length, 20 mm width and 4 mm thickness.

The tensile properties of the filled/unfilled PTFE samples were measured by a Zwick Z250 universal tester equipped with a 20 kN load cell (EN ISO 527-2). The crosshead speed was 10 mm/min until 0.5% strain and 100 mm/min after 0.5% strain. The dumbbell-shaped sample size was 80 mm total length, 60 mm neck length, 5 mm neck width, and 4 mm thickness.

Related to the hardness, compressive, shear and tensile tests, the mean and standard deviation values were calculated from at least five measurements.

#### 2.3.5. Wear Tests

The tribological characterisation was performed with a Wazau TRM 1000 tribometer from Dr.-Ing. Georg Wazau Mess- und Prüfsysteme GmbH, Germany. The applied configuration was pin-on-disc with excentrically rotating cylindrical polymer pin and stationary steel disc counterface. The counterface material was 42CrMo4. All disc counterfaces were surface finished by turning to nominal Ra 0.3 µm average surface roughness in a spiral pattern. The investigated polymer pin samples had a diameter of 8 mm with 5 mm thickness (length), while the counterfaces had a diameter of 50 mm. The wear track centreline on the steel discs was at 30 mm diameter. The applied sliding speed was 61 rpm (corresponding to a circumferential linear speed of 0.1 m/s), while the applied normal force was 151 N (corresponding to 3 MPa contact pressure). The total sliding distance per test was set at 1000 m. The mean and standard deviation values were calculated from at least 5 measurements. The specific wear rate of polymer samples was calculated after wear testing by using Equation (1):

(1) $$k=\frac{\Delta m}{\rho \cdot F_{N}\cdot d_{s}},$$

where k is the specific wear rate (mm^3^/Nm), Δm is the measured mass loss (g) by a weight balance after wear test, ρ is the density of the pin sample (g/mm^3^), $F_{N}$ is the applied normal force (N) measured by the tribometer, and $d_{s}$ is the sliding distance (m) calculated by the tribometer.

## 3. Results and Discussion

### 3.1. Micro-analysis of the Samples and the Applied Mechanical Stirring

The freeze-fractured (cryo-fractured) surfaces of PTFE/graphene-4, PTFE/Al_2_O_3_-4, PTFE/BA80-4 and PTFE/MG70-4 samples were analysed by Raman spectroscopy or EDS. The samples were fractured after being cooled down in liquid nitrogen (−196 °C).

#### 3.1.1. Dispersion of Graphene

**Figure 1:**
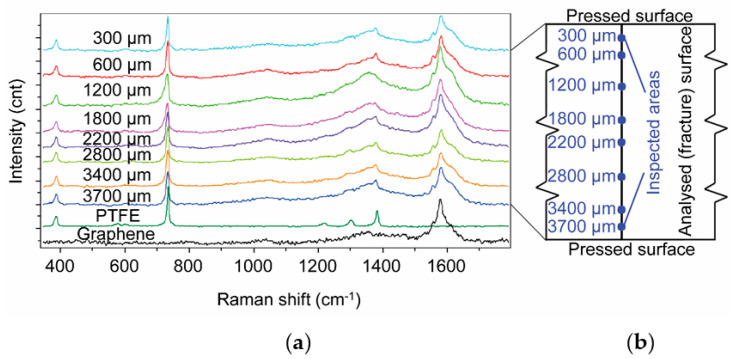
(**a**): Raman spectra of PTFE/graphene-4 sample in different depth on the cryo-fractured surface; (**b**): the investigated locations in PTFE/graphene-4. (The reference spectra of the neat graphene and PTFE are introduced at the bottom of the diagram).

The Raman spectra of PTFE/graphene-4 can be seen in Figure 1a. The graphene-filled sample was fractured after cooling down with liquid nitrogen. The size of the analysed area in one measurement point is 50 µm × 50 µm.

The graphene dispersion was checked in eight locations at different depths from the pressed surface (Figure 1b). In all measured areas of the cryo-fractured surface, there is a significant peak close to 1600 cm^−1^, which comes from the graphene content of the area. These spectra indicate that there was no significant graphene migration or diffusion from the core to the edge (pressed surface of the sample) neither at the room temperature pressing nor at sintering temperature.

#### 3.1.2. Dispersion of Alumina (Al_2_O_3_)

**Figure 2:**
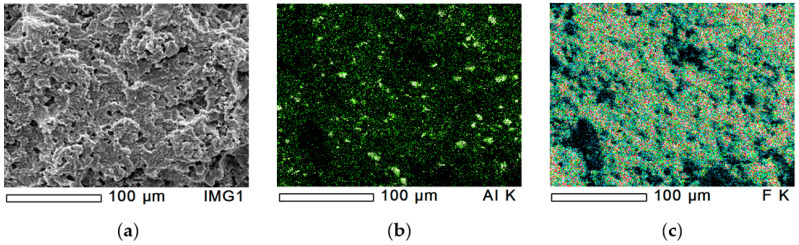
EDS analysis of PTFE/Al_2_O_3_-4 sample with 500× magnification. (**a**): original cryo-fractured surface, (**b**): aluminium content and (**c**): fluorine content.

**Figure 3:**
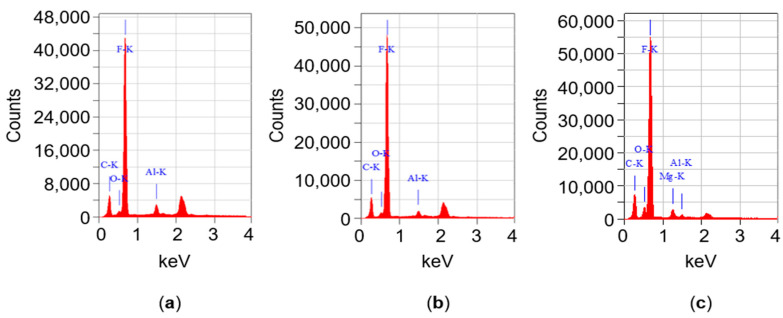
EDS spectra of PTFE/Al_2_O_3_-4 (**a**), PTFE/BA80-4 (**b**) and PTFE/MG70-4 (**c**) samples. The unmarked peak (~2.1–2.2 keV) is related to the sputtered gold (Au) on the sample surfaces.

The dispersion of Al_2_O_3_-filler in PTFE/Al_2_O_3_-4 was analysed by EDS mapping at four different locations as a function of depth from the pressed surface. Figure 2a shows the original cryo-fractured surface, while Figure 2b,c introduce the aluminium and fluorine content, respectively. The fluorine atoms come from the PTFE backbone. Figure 2b,c have black spots in the same positions, with similar shape and size. From these areas, insufficient number of reflected X-ray photons reached the detector caused by surface topology (Figure 2a). The dispersion of aluminium realised by the rotating blade grinder is shown in Figure 2b. Further EDS analyses were performed at three other locations of the cryo-fractured surface. All inspections showed similar results and similar dispersion. Besides the larger particles in the range of 10 µm, finer homogeneously dispersed Al_2_O_3_-powder also can be seen. The size of these finer filler particles was below 1 µm. The significant aluminium peak (Al-K, where K represents the K electron shell) was also shown by the EDS spectrum (Figure 3a). The total detected percentage of aluminium was 3.77%, 3.89%, 4.32%, and 3.39% at the four locations, which means that the dispersion effect of the dry mechanical stirring was successful at macro-level.

#### 3.1.3. Dispersion of Boehmite Alumina (BA80, AlO(OH))

**Figure 4:**
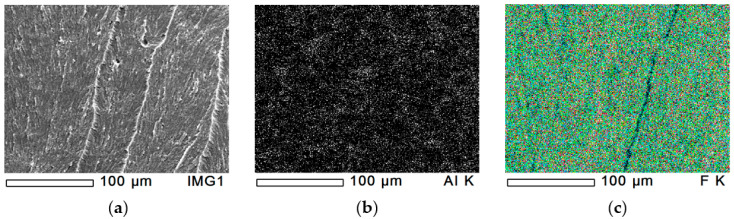
EDS analysis of PTFE/BA80-4 sample with 500× magnification. (**a**): original cryo-fractured surface, (**b**): aluminium content and (**c**): fluorine content.

The dispersion of BA80-filler in PTFE/BA80-4 was also analysed by EDS, and it is presented in Figure 4. Figure 4a–c displays the cryo-fractured surface and the aluminium and fluorine content, respectively. The average size of the filler agglomerates was below 1 µm. The presence of an aluminium peak (Al-K, where K represents the K electron shell) was significant in the EDS spectra (Figure 3b). The total percentage of aluminium was 2.73%, 2.17%, 2.53%, and 2.09% at the four locations, which indicates efficient dispersion of the fine filler powder.

#### 3.1.4. Dispersion of Hydrotalcite (MG70)

**Figure 5:**
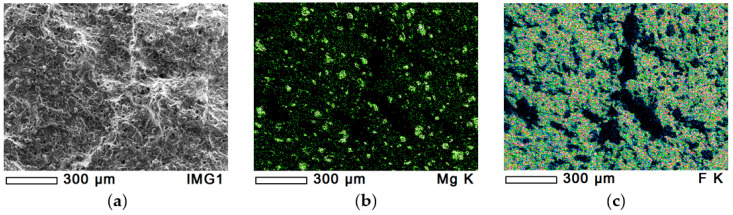
EDS analysis of PTFE/MG70-4 sample with 100× magnification. (**a**): original cryo-fractured surface, (**b**): magnesium content and (**c**): fluorine content.

The dispersion of MG70-filler in PTFE/MG70-4 is presented in Figure 5. Figure 5a–c shows the cryo-fractured surface and the magnesium and fluorine content, respectively. The dispersion of magnesium provided by the rotating blade grinder can be seen in Figure 5b. Similarly to Figure 2, Figure 5b,c have black spots from where insufficient number of reflected X-ray photons reached the detector. From the given cryo-fractured surface, four EDS tests were performed, all of them with similar results. The magnesium peak (MG-K, where K represents the K electron shell) was significant in the EDS spectra (Figure 3c). The total percentage of magnesium was 2.65%, 3.02%, 2.68%, and 3.10%. All EDS mappings indicate that the dispersion of MG70-filler was efficient.

### 3.2. Density of the Unfilled/Filled PTFE Materials

The density of neat PTFE, PTFE/graphene, PTFE/Al_2_O_3_, PTFE/BA80, and PTFE/MG70 materials can be seen in Table 2. The reference (neat) PTFE had 2.17 g/cm^3^ density. In the case of graphene-filled samples, the density decreased as the filler content increased. This is caused by the low density of graphene filler. The density of PTFE/graphene-16 sample was only 1.95 g/cm^3^, which is ~10% lower compared to neat PTFE. Both Al_2_O_3_ and BA80 increased the density compared to neat PTFE, due to the higher density of alumina and boehmite alumina. These increases were observed as slight changes in density. The lower density of PTFE/MG70 samples is supposed to come from the decomposition of the functional groups of MG70 filler during the sintering process. During decomposition of MG70-filler, some of the produced gas cannot escape from the PTFE, increasing the final material porosity and thus lowering its density.

### 3.3. Thermal Conductivity of the Unfilled/Filled PTFE Materials

The thermal conductivity of unfilled and filled PTFE materials can be seen in Table 2 with the mean and standard deviation values. Neat PTFE had a thermal conductivity of 0.24 (W/mK). Graphene in 4/8/16 wt % filler content increased the thermal conductivity with ~29%/~84%/~157%, respectively. The excellent thermal properties of graphene cause this phenomenon. This higher thermal conductivity can be beneficial e.g., in wear process as more frictional heat can be removed from the contact surface. In this way, the surface temperature is decreasing, which results in a smaller reduction in the mechanical properties of the polymer. The tendency of the thermal conductivity of graphene-filled PTFE materials is in agreement with the experimental and computational results of Z. Jin et al. [19]. Al_2_O_3_-, BA80- and MG70-fillers only slightly increased the thermal conductivity.

### 3.4. Mechanical Analyses of the Unfilled/Filled PTFE Materials

#### 3.4.1. DMA Analysis

**Figure 6:**
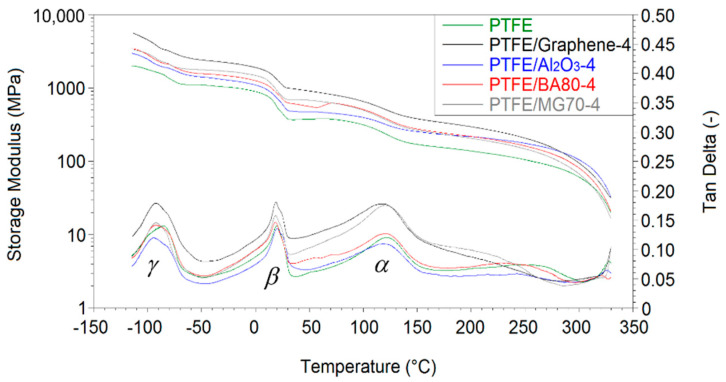
DMA characterisation of neat PTFE and 4 wt % filled PTFE samples.

DMA analysis was performed for neat PTFE and for all filled PTFE samples with 4 wt % filler content. The storage modulus and tangent delta (loss factor) as a function of temperature can be seen in Figure 6. The storage moduli of the materials at −50, 20 and 150 °C temperature are presented in Table 3.

In agreement with the literature [14], all filled PTFE samples had markedly higher storage moduli with respect to the reference unfilled PTFE. Graphene, Al_2_O_3_, BA80 and MG70 improved the storage modulus at 20 °C by ~138%, ~33%, ~54%, and ~67%, respectively (Table 3). One possible reason for this improvement can be the restriction of the molecular chain motion due to the filler particles. Three main steps in the storage modulus curve and three main peaks on the loss factor (tangent delta) curve can be observed in Figure 6. The three significant peaks of tangent delta can be found at −95–85 °C, 18–20 °C and 117–121 °C temperature, depending on the given material (Figure 6 and Table 3). This is in agreement with the literature [20]. The temperature peaks correspond to phase transitions of PTFE: The first peak is the γ-transition, the second one is the β-transition while the third one is the α-transition [20]. Significant difference in the α/β-transition peak temperature in the function of the applied filler was not found.

#### 3.4.2. Hardness

The hardness values of the sintered materials can be seen in Table 4. The reference unfilled PTFE had 54.3 (Shore-D) hardness. The developed graphene-filled materials had slightly higher hardness compared to neat PTFE, except for 16 wt % filler content where the hardness was slightly lower. Al_2_O_3_ and BA80 filled materials reached a higher hardness, and this value was increased as the filler content increased. The highest hardness was measured in PTFE/BA80-16, PTFE/BA80-8 and PTFE/Al_2_O_3_-4 samples, as 60.0, 59.1 and 58.8 (Shore-D) respectively. PTFE/MG70-1 and PTFE/MG70-4 samples had the same values as the reference unfilled PTFE.

#### 3.4.3. Compressive Properties

**Figure 7:**
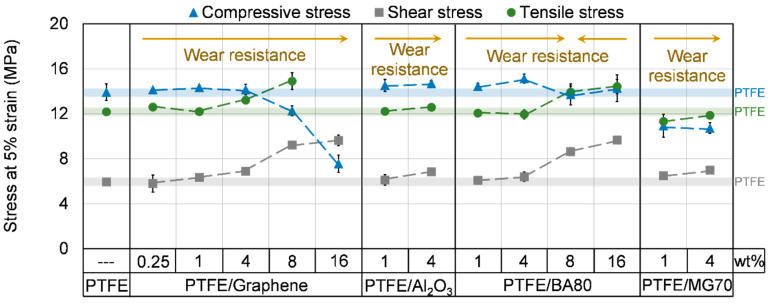
Compressive, shear and tensile stress of unfilled and filled PTFE samples at 5% strain. The blue, grey and green transparent lines display the compressive, shear and tensile stress of neat PTFE, respectively.

**Figure 8:**
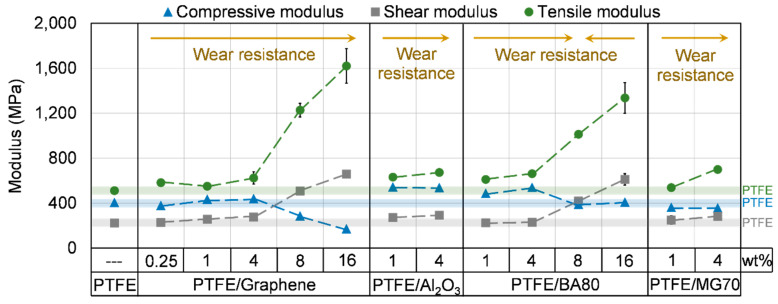
Compressive, shear and tensile modulus of unfilled and filled PTFE samples. The blue, grey and green transparent lines display the compressive, shear and tensile modulus of neat PTFE, respectively.

The compressive properties of the sintered samples are introduced in Figure 7 and Figure 8, Table 4 and Table 5. PTFE/Al_2_O_3_-1 and PTFE/Al_2_O_3_-4 samples did not reach significantly higher compressive stress compared to neat PTFE, while their compressive modulus increased remarkably. MG70 filler decreased both the compressive stress and modulus. Graphene with low filler content (0.25, 1 and 4 wt %) did not change the compressive properties remarkably, while BA80 in 1 and 4 wt % increased the compressive modulus significantly compared to neat PTFE. In higher filler content, BA80 is observed as a superior additive compared to graphene filler. Samples with 8 and 16 wt % BA80 reached the same level of compressive modulus as neat PTFE, while the application of graphene in higher percentages decreased both compressive stress and compressive modulus significantly. This is attributed to the low density of graphene, which resulted in a high volume fraction of graphene in the developed PTFE/Graphene-8 and PTFE/Graphene-16 samples. PTFE/Al_2_O_3_-1, PTFE/BA80-4 and PTFE/Al_2_O_3_-4 samples reached the highest improvement in compressive modulus which was ~34.2%, 33.1% and 32.6% higher compared to neat PTFE, respectively.

As it is introduced in Section 3.4.1, regarding the β-transition of PTFE, the peak temperature can be found around 19–20 °C. Between 0 and 50 °C, a slope in the storage modulus graph is registered, which caused a significant decrease in these modulus values. As the peak temperature of β-transition is relatively close to room temperature (23 °C) where all of the static mechanical tests were done, further compressive tests were carried out at 50 °C with 4 wt % filler content samples (Table 5). As it is expected from DMA, the compressive stress and modulus values were lower at 50 °C. An important conclusion is that the tendencies of the measured compressive properties are similar in the case of both room temperature and 50 °C, which is in agreement with the DMA results (Figure 6).

#### 3.4.4. Shear Properties

**Figure 9:**
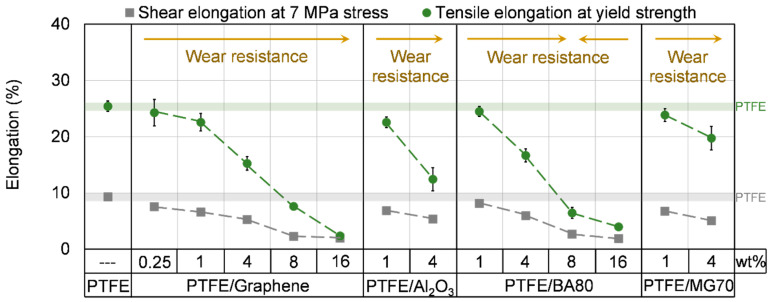
Shear and tensile elongation of unfilled and filled PTFE samples. The grey and green transparent lines display the shear and tensile elongation of neat PTFE, respectively.

The shear properties of the unfilled and filled PTFE samples are introduced in Figure 7, Figure 8 and Figure 9 and in Table 6. All of the filler increased the shear stress at 2% and 5% strain and the shear modulus as the filler content increased. In agreement with the observed changes in the stress and modulus values, the elongation of the samples decreased as the filler content increased. A possible reason for this phenomenon is that due to the applied fillers the movement of the PTFE molecular chains is restricted, which results in an increased modulus and decreased elongation. The higher shear modulus confirms the changes in storage modulus measured by DMA (Figure 6). Because the elongation of PTFE-based samples was so high and given the limited displacement range of the tensile tester, it was not possible to reach a local maximal value for shear stress. In this way, the elongation was compared at 7 MPa stress, as this level of shear stress was reached by all of the tested samples (Figure 9). BA80 and graphene in high filler content (8 and 16 wt %) have modified the shear properties at the same level. PTFE/BA80-16, PTFE/Graphene-16, PTFE/BA80-8, and PTFE/Graphene-8 samples had the lowest elongation, 1.86%, 1.95%, 2.67%, and 2.29% (Figure 9), respectively, while their shear modulus was the highest, 611.4, 657.4, 418.9, and 507.8 MPa (Figure 8), respectively. In comparison, neat PTFE had 9.28% elongation at 7 MPa stress and 223.2 MPa shear modulus.

#### 3.4.5. Tensile Properties

The tensile properties of the unfilled and filled PTFE samples are introduced in Figure 7, Figure 8 and Figure 9 and in Table 7 and Table 8. The tendencies of the measured tensile stress at 2% and 5% strain; the tensile modulus and the elongation at yield strength are in agreement with the shear properties and the results of DMA tests (Figure 6). In case of neat PTFE 512 MPa tensile modulus, 25.4% elongation at yield strength and 288% elongation at break was recorded. Compared to the reference neat PTFE, PTFE/Graphene-16 and PTFE/BA80-16 samples reached ~216% and ~161% improvement in tensile modulus (Figure 8), which can be explained again with the restricted movement of the long PTFE molecular chains. Another explanation for the higher modulus can be the aggregation of nanoparticles, which have a stiffness-increasing mechanism [21]. Focusing on BA80 filler, the tensile modulus increased as the filler content increased, this tendency confirms the results of Pedrazzoli et al., who investigated boehmite alumina filler in polypropylene matrix [15]. The tensile modulus enhancement achieved by graphene filler is also in agreement with the literature [22]. PTFE/Graphene-16 and PTFE/BA80-16 specimens had one order of magnitude lower elongation at yield strength compared to neat PTFE (Figure 9). Remarkably tendencies in yield strength were not registered. Compared to the reference neat PTFE, PTFE/Graphene-8 and PTFE/Graphene-16 samples decreased the stress at break significantly, and their elongation at break was one and two orders of magnitude lower, respectively. When the elongation at break for BA80 filled specimens is considered with respect to the reference neat PTFE, it can be stated that the elongation is increased, which is in agreement with the literature [16].

### 3.5. Wear Behaviour of the Developed Unfilled/Filled PTFE Materials

The calculated specific wear rates of the tested unfilled/filled PTFE samples are presented in Table 9. Neat PTFE had 5.16 × 10^−4^ (mm^3^/Nm) wear rate. The lowest wear rate was reached with PTFE/Al_2_O_3_-4 polymer samples with a value of 2.91 × 10^−6^ (mm^3^/Nm), which is more than two orders of magnitude improvement compared to the reference neat PTFE. It is in agreement with the results of Krick et al. who also reached two to three orders of magnitude enhancement in wear resistance with the use of 5 wt % alumina in PTFE matrix [9]. PTFE/Al_2_O_3_-1 had ~82% reduction in wear rate compared to unfilled PTFE. Graphene in 4/8/16 wt % filler content also significantly decreased the wear; the reduction was around one to two orders of magnitude with a value of 4.72 × 10^−5^, 3.17 × 10^−5^ and 8.51 × 10^−6^ (mm^3^/Nm), respectively. Kandanur et al. also registered a tendency with increasing wear resistance as the graphene filler content increased [11]. The wear rate of PTFE/graphene-0.25 and PTFE/graphene-1 was similar to the reference PTFE. PTFE/BA80 in 1/4/8/16 wt % filler content decreased the wear rate of neat PTFE by 53%, 61%, 86%, and 78%, respectively. MG70 additive showed similar wear rate as the reference PTFE, which comes from its high decomposition during sintering temperature (370 °C). The wear rate improvements achieved by graphene and Al_2_O_3_ fillers are in agreement with the literature [9,10,11].

**Figure 10:**
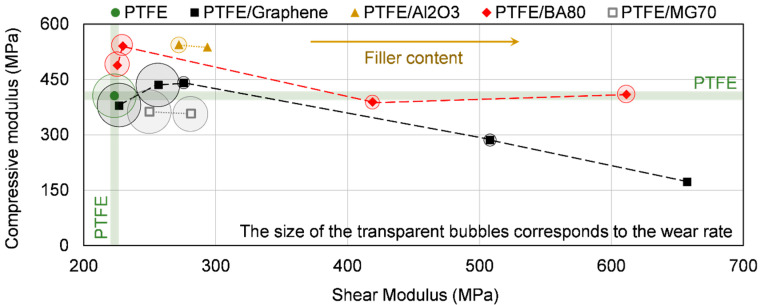
Compressive modulus as a function of the shear modulus of unfilled and filled PTFE samples. The size of the bubbles correlates to the wear rate of the samples.

The wear rate as a function of the shear and compressive modulus is depicted in Figure 10. It can be seen that no clear relation exists between the compressive/shear modulus and the wear rate. PTFE/Al_2_O_3_-4 material had the lowest wear rate, with a low shear modulus and high compressive modulus compared to the other PTFE-based materials. In contrast with this, PTFE/graphene–16 had the second lowest wear rate, but it has the highest shear modulus and the lowest compressive modulus. The low wear rate of PTFE/Al_2_O_3_-4 material can neither come from its thermal conductivity, hardness or tensile modulus, as these properties only slightly changed compared to neat PTFE. It means that focusing on the wear rate, the dominating factor can only be the type of the applied fillers (e.g., filler material, particle size and geometry) and in this way the transfer layer formation. The applied filler and, consequently, the transfer layer formation can have a dominating (primary) role in the wear mechanism of the investigated materials.

It is also important to mention that the higher tensile/shear modulus and thermal conductivity can also affect the wear resistance of the materials. E.g., the higher thermal conductivity can be beneficial in wear process as more frictional heat can be removed from the contact surface. As a result, the surface temperature of the polymer is decreasing, which results in a smaller reduction in the mechanical properties of the polymer. Focusing on graphene filler, it can be seen that the wear rate was decreased as the filler content increased, and simultaneously the tensile/shear modulus and thermal conductivity were increased as the filler content increased. In opposition to this, in the case of BA80 filler, the wear rate was not remarkably influenced by the increased filler content and accompanying increased shear and tensile modulus. As a conclusion, it can be stated that the shear/tensile properties and thermal conductivity have only a secondary role in the wear mechanism of the investigated materials.

## 4. Conclusions

In this research, the physical, thermal, viscoelastic, mechanical, and wear analysis of neat PTFE and graphene, alumina, boehmite alumina, and hydrotalcite-filled PTFE were introduced.

- The applied blending method was an intensive dry mechanical stirring, which is suitable to make homogeneous blends. The homogeneity of the powder blends was investigated by EDS/Raman spectrometry.
- Graphene filler due to its excellent thermal properties remarkably increased the thermal conductivity of PTFE-based samples. Compared to neat PTFE, graphene in 4/8/16 wt % improved the thermal conductivity with ~29%/~84%/~157%, respectively.
- Compared to neat PTFE, the shear and tensile modulus of the developed mono-filled samples were increased together with the increase of the filler content. It is in line with the changes in storage modulus measured by DMA tests. All the applied fillers increased the storage modulus of PTFE. In agreement with the changes observed for the shear, tensile and storage modulus, the elongation at yield strength and the measured elongation during shear tests were significantly reduced with decreasing filler content. This reduced ductility, and the increase of the modulus values can be explained based on the restricted molecular chain motion caused by the applied fillers.
- Focusing on the compressive properties, with higher filler content (8 and 16 wt %), boehmite alumina is observed as a superior additive compared to graphene filler. Graphene significantly decreased the compressive stress and modulus compared to neat PTFE. Samples with lower alumina or boehmite content remarkably increased the compressive modulus.
- As the peak temperature of β-transition of PTFE is close to room temperature (19–20 °C), compressive tests at 50 °C were carried out as a confirmation of the measurements run at room temperature. The tendencies of the registered compressive stress and modulus values between room temperature (23 °C) and 50 °C are close to each other. It means that at the temperature range of the β-transition of PTFE, the ratio of the mechanical performance of the measured specimens does not change remarkably. It is in agreement with the measured storage modulus of the samples.
- PTFE/Al_2_O_3_-4 polymer samples had the lowest wear rate, reaching more than two orders of magnitude improvement compared to the neat PTFE. This ultra-low wear rate is not induced by the modified thermal conductivity, hardness or compressive/shear/tensile modulus as these values only slightly changed compared to the neat PTFE. It is supposed that this improvement comes from the modified transfer layer formation.
- The type of filler has a dominating (primary) role in wear mechanism. The increased shear/tensile properties and thermal conductivity have a lower influence on the wear mechanism (secondary role) in case of the investigated PTFE-based materials.
- The developed alumina and graphene-filled PTFE materials can be used in a wide field of tribological applications, e.g., as sliding bearings or seals. Focusing on the wear results, the suggested compositions are 4 wt % alumina or 4–16 wt % graphene, as these materials reached more than one order of magnitude enhancement in wear resistance.

## Figures and Tables

**Table 1:** The developed neat PTFE and PTFE-based materials.

Materials	Matrix	Filler	Filler Content (wt %)
PTFE	PTFE	---	---
PTFE/graphene-0.25	PTFE	Graphene	0.25
PTFE/graphene-1	PTFE	Graphene	1
PTFE/graphene-4	PTFE	Graphene	4
PTFE/graphene-8	PTFE	Graphene	8
PTFE/graphene-16	PTFE	Graphene	16
PTFE/Al_2_O_3_-1	PTFE	Alumina (Al_2_O_3_)	1
PTFE/Al_2_O_3_-4	PTFE	Alumina (Al_2_O_3_)	4
PTFE/BA80-1	PTFE	Boehmite alumina (BA80)	1
PTFE/BA80-4	PTFE	Boehmite alumina (BA80)	4
PTFE/BA80-8	PTFE	Boehmite alumina (BA80)	8
PTFE/BA80-16	PTFE	Boehmite alumina (BA80)	16
PTFE/MG70-1	PTFE	Hydrotalcite (MG70)	1
PTFE/MG70-4	PTFE	Hydrotalcite (MG70)	4

**Table 2:** Average density and thermal conductivity of sintered PTFE materials. The standard deviation of the density was less than ± 0.003 (g/cm^3^) for all of the samples.

Samples	Density (g/cm^3^)	Thermal Conductivity (W/mK)
PTFE	2.17	0.24 ± 0.01
PTFE/graphene-0.25	2.17	0.25 ± 0.01
PTFE/graphene-1	2.17	0.25 ± 0.02
PTFE/graphene-4	2.14	0.31 ± 0.01
PTFE/graphene-8	2.07	0.45 ± 0.02
PTFE/graphene-16	1.95	0.62 ± 0.03
PTFE/Al_2_O_3_-1	2.18	0.25 ± 0.02
PTFE/Al_2_O_3_-4	2.20	0.27 ± 0.02
PTFE/BA80-1	2.17	0.25 ± 0.02
PTFE/BA80-4	2.19	0.27 ± 0.02
PTFE/BA80-8	2.19	0.31 ± 0.01
PTFE/BA80-16	2.20	0.33 ± 0.00
PTFE/MG70-1	2.14	0.26 ± 0.01
PTFE/MG70-4	2.13	0.28 ± 0.01

**Table 3:** DMA analysis of sintered filled/unfilled PTFE specimens.

	**Storage Modulus,**	**Storage Modulus,**	**Storage Modulus,**
	**MPa (−50 °C)**	**MPa (20 °C)**	**MPa (150 °C)**
PTFE	1102	545	170
PTFE/graphene-4	2412	1297	381
PTFE/Al_2_O_3_-4	1413	723	254
PTFE/BA80-4	1567	842	277
PTFE/MG70-4	1773	908	266
	**Peak Temperature (γ)**	**Peak Temperature (β)**	**Peak Temperature (α)**
	**(°C)**	**(°C)**	**(°C)**
PTFE	−85.4	19.7	121.3
PTFE/graphene-4	−92.9	18.9	117.0
PTFE/Al_2_O_3_-4	−94.9	20.1	117.9
PTFE/BA80-4	−92.5	18.5	120.7
PTFE/MG70-4	−92.3	18.6	119.9

**Table 4:** Average Shore-D hardness, compressive stress at 5% and 10% strain and average compressive modulus of the sintered materials.

Samples	Hardness (Shore-D)	Compressive Stress at 5% Strain, MPa	Compressive Stress at 10% Strain, MPa	Compressive Modulus, MPa
PTFE	54.3 ± 0.9	13.92 ± 0.73	18.91 ± 0.57	405.9 ± 11.8
PTFE/graphene-0.25	55.0 ± 0.2	14.14 ± 0.19	19.51 ± 0.15	378.6 ± 9.1
PTFE/graphene-1	55.7 ± 0.9	14.33 ± 0.21	19.36 ± 0.25	435.2 ± 14.8
PTFE/graphene-4	55.6 ± 1.2	14.15 ± 0.48	19.56 ± 0.24	440.2 ± 17.8
PTFE/graphene-8	55.3 ± 0.7	12.27 ± 0.45	18.37 ± 0.13	286.0 ± 10.9
PTFE/graphene-16	53.9 ± 0.9	7.57 ± 0.77	14.35 ± 0.35	172.2 ± 9.9
PTFE/Al_2_O_3_-1	57.7 ± 1.0	14.52 ± 0.53	19.03 ± 0.64	544.7 ± 11.0
PTFE/Al_2_O_3_-4	58.8 ± 0.4	14.70 ± 0.26	19.11 ± 0.29	538.2 ± 10.4
PTFE/BA80-1	56.3 ± 0.7	14.44 ± 0.27	18.98 ± 0.32	488.3 ± 10.9
PTFE/BA80-4	58.4 ± 0.5	15.12 ± 0.41	19.55 ± 0.44	540.4 ± 16.1
PTFE/BA80-8	59.1 ± 0.9	13.72 ± 0.93	20.10 ± 0.34	388.9 ± 9.2
PTFE/BA80-16	60.0 ± 1.1	14.27 ± 1.19	21.79 ± 0.26	409.2 ± 9.8
PTFE/MG70-1	53.7 ± 0.9	10.94 ± 1.01	17.16 ± 0.12	362.5 ± 9.3
PTFE/MG70-4	54.5 ± 0.7	10.73 ± 0.48	16.69 ± 0.24	357.4 ± 13.9

**Table 5:** Average compressive stress at 5% and 10% strain and average compressive modulus of the sintered materials at 50 °C.

Samples	Compressive Stress at 5% Strain, MPa (50 °C)	Compressive Stress at 10% Strain, MPa (50 °C)	Compressive Modulus, MPa (50 °C)
PTFE	11.13 ± 0.27	16.00 ± 1.34	318.4 ± 12.7
PTFE/graphene-4	11.00 ± 0.27	16.39 ± 0.17	351.0 ± 7.7
PTFE/Al_2_O_3_-4	12.47 ± 0.12	17.06 ± 0.19	422.5 ± 17.3
PTFE/BA80-4	12.02 ± 0.55	16.53 ± 0.19	422.0 ± 13.0
PTFE/MG70-4	11.21 ± 0.62	15.99 ± 0.09	325.9 ± 10.0

**Table 6:** Average shear stress at 2% and 5% strain, average elongation at 7 MPa stress and average shear modulus of the sintered materials.

Samples	Shear Stress at 2% Strain, MPa	Shear Stress at 5% Strain, MPa	Elongation at 7 MPa stress, %	Shear Modulus, MPa
PTFE	3.84 ± 0.08	5.93 ± 0.09	9.28 ± 0.55	223.2 ± 15.1
PTFE/graphene-0.25	3.85 ± 0.64	5.79 ± 0.76	7.55 ± 0.31	226.8 ± 5.3
PTFE/graphene-1	3.94 ± 0.20	6.35 ± 0.31	6.59 ± 0.41	256.6 ± 21.0
PTFE/graphene-4	4.44 ± 0.26	6.89 ± 0.28	5.26 ± 0.60	275.8 ± 25.8
PTFE/graphene-8	6.58 ± 0.22	9.23 ± 0.22	2.29 ± 0.16	507.8 ± 19.6
PTFE/graphene-16	7.11 ± 0.33	9.63 ± 0.47	1.95 ± 0.22	657.4 ± 8.2
PTFE/Al_2_O_3_-1	4.22 ± 0.02	6.12 ± 0.47	6.82 ± 0.24	272.1 ± 25.9
PTFE/Al_2_O_3_-4	4.42 ± 0.31	6.83 ± 0.17	5.36 ± 0.39	293.7 ± 11.2
PTFE/BA80-1	3.75 ± 0.38	6.08 ± 0.01	8.16 ± 0.54	225.5 ± 16.0
PTFE/BA80-4	4.19 ± 0.35	6.40 ± 0.42	5.95 ± 0.07	229.6 ± 2.7
PTFE/BA80-8	6.07 ± 0.30	8.63 ± 0.35	2.67 ± 0.32	418.9 ± 28.1
PTFE/BA80-16	7.26 ± 0.34	9.68 ± 0.33	1.86 ± 0.21	611.4 ± 50.3
PTFE/MG70-1	4.35 ± 0.43	6.49 ± 0.22	6.73 ± 0.59	249.9 ± 38.2
PTFE/MG70-4	4.79 ± 0.32	6.99 ± 0.26	5.05 ± 0.68	281.3 ± 30.0

**Table 7:** Average tensile stress at 2% and 5% strain and average tensile modulus of the sintered materials.

Samples	Tensile Stress at 2% Strain, MPa	Tensile Stress at 5% Strain, MPa	Tensile Modulus, MPa
PTFE	10.16 ± 0.18	12.18 ± 0.14	511.6 ± 22.3
PTFE/graphene-0.25	10.34 ± 0.13	12.60 ± 0.08	582.6 ± 10.0
PTFE/graphene-1	10.29 ± 0.14	12.19 ± 0.21	551.0 ± 5.5
PTFE/graphene-4	11.23 ± 0.15	13.21 ± 0.18	624.1 ± 53.8
PTFE/graphene-8	12.22 ± 0.82	14.90 ± 0.75	1227.4 ± 60.3
PTFE/graphene-16	13.31 ± 1.32	---	1620.5 ± 153.4
PTFE/Al_2_O_3_-1	10.33 ± 0.18	12.22 ± 0.24	632.0 ± 12.5
PTFE/Al_2_O_3_-4	11.51 ± 0.06	12.58 ± 0.11	673.4 ± 23.5
PTFE/BA80-1	10.52 ± 0.43	12.06 ± 0.27	611.2 ± 7.8
PTFE/BA80-4	10.81 ± 0.45	11.95 ± 0.40	661.9 ± 11.2
PTFE/BA80-8	11.87 ± 0.41	13.92 ± 0.55	1013.1 ± 29.2
PTFE/BA80-16	13.27 ± 0.47	14.42 ± 0.63	1336.1 ± 136.1
PTFE/MG70-1	9.58 ± 0.22	11.33 ± 0.26	538.8 ± 13.2
PTFE/MG70-4	10.64 ± 0.32	11.86 ± 0.33	699.8 ± 12.5

**Table 8:** Average Yield stress, elongation at yield strength, tensile stress at break, and elongation at break of the sintered materials.

Samples	Yield Strength, MPa	Elongation at Yield Strength, %	Tensile Stress at Break, MPa	Elongation at Break, %
PTFE	12.69 ± 0.12	25.39 ± 0.93	20.06 ± 1.26	288.0 ± 15.3
PTFE/graphene-0.25	14.52 ± 0.19	24.26 ± 2.36	24.81 ± 2.99	348.8 ± 35.9
PTFE/graphene-1	12.67 ± 0.28	22.55 ± 1.57	23.35 ± 1.34	356.9 ± 67.0
PTFE/graphene-4	13.32 ± 0.21	15.24 ± 1.20	17.15 ± 0.74	226.0 ± 22.1
PTFE/graphene-8	15.17 ± 0.74	7.60 ± 0.26	13.05 ± 1.35	15.4 ± 0.8
PTFE/graphene-16	13.06 ± 0.95	2.36 ± 0.38	13.69 ± 1.51	2.5 ± 0.4
PTFE/Al_2_O_3_-1	12.60 ± 0.20	22.55 ± 0.96	24.11 ± 2.22	369.9 ± 3.0
PTFE/Al_2_O_3_-4	12.60 ± 0.13	12.43 ± 2.06	20.17 ± 1.72	306.5 ± 25.8
PTFE/BA80-1	12.56 ± 0.22	24.46 ± 0.88	22.32 ± 1.31	378.2 ± 81.2
PTFE/BA80-4	12.21 ± 0.29	16.66 ± 1.15	22.05 ± 1.68	344.7 ± 43.9
PTFE/BA80-8	13.98 ± 0.56	6.44 ± 0.96	23.16 ± 0.53	368.2 ± 8.7
PTFE/BA80-16	14.51 ± 0.62	3.96 ± 0.24	17.50 ± 0.95	320.0 ± 13.0
PTFE/MG70-1	11.93 ± 0.27	23.84 ± 1.13	21.78 ± 1.58	319.6 ± 36.2
PTFE/MG70-4	12.05 ± 0.38	19.73 ± 2.11	23.40 ± 2.36	438.9 ± 73.6

**Table 9:** The measured wear rates of the tested unfilled/filled PTFE.

Materials	Wear Rate (mm^3^/mN)	
	Average	Deviation (%)
PTFE	5.16 × 10^−4^	8.8
PTFE/graphene-0.25	5.47 × 10^−4^	6.0
PTFE/graphene-1	5.07 × 10^−4^	20.6
PTFE/graphene-4	4.72 × 10^−5^	17.2
PTFE/graphene-8	3.17 × 10^−5^	25.6
PTFE/graphene-16	8.51 × 10^−6^	39.6
PTFE/Al_2_O_3_-1	9.26 × 10^−5^	12.5
PTFE/Al_2_O_3_-4	2.91 × 10^−6^	68.4
PTFE/BA80-1	2.40 × 10^−4^	10.9
PTFE/BA80-4	2.01 × 10^−4^	6.7
PTFE/BA80-8	7.27 × 10^−5^	10.3
PTFE/BA80-16	1.12 × 10^−4^	10.9
PTFE/MG70-1	5.39 × 10^−4^	2.2
PTFE/MG70-4	4.49 × 10^−4^	12.7
